# Improved piezocatalytic performance of BaTiO₃ nanowires via in situ pore structure regulation

**DOI:** 10.1186/s40580-025-00514-0

**Published:** 2025-10-28

**Authors:** Yiren Liu, Qinghu Guo, Zhonghua Yao, Hanxing Liu, Shujun Zhang, Hua Hao

**Affiliations:** 1 State Key Laboratory of Silicate Materials for Architectures, State Key Laboratory of Advanced Technology for Materials Synthesis and Processing, School of Material Science and Engineering, International School of Material Science and Engineering, Wuhan University of Technology, Wuhan, 430070 China; 2 National Energy Key Laboratory for New Hydrogen-Ammonia Energy Technologies, Foshan Xianhu Laboratory, Foshan, 528200 China; 3 Institute for Superconducting and Electronic Materials, Faculty of Engineering and Information Sciences, University of Wollongong, Wollongong, NSW 2500 Australia; 4 Department of Chemistry, City University of Hong Kong, Kowloon, 999077 Hong Kong China

**Keywords:** Piezocatalysis, BaTiO_3_, Nanowires, Pore structure

## Abstract

In conventional catalytic reactions, the specific surface area of the catalyst plays a pivotal role in determining the reaction rate. Similarly, in piezocatalysis, the modulation of pore structure is equally critical. In this study, BaTiO₃ nanowires were synthesized via a two-step solvothermal process, wherein the pore architecture of the catalyst was engineered by adjusting the reaction solvent and other parameters. This structural optimization not only facilitated the transport of reactants and enhanced the bulk resistivity, but also induced an internal electric field within the catalyst. Consequently, the screening charges generated by the piezoelectric effect were more effectively localized on the sample surface to engage in redox reactions, thereby yielding a highly active piezocatalyst. The optimized catalyst achieved a degradation efficiency of 98% for Rhodamine B (RhB) solution within 10 min, accompanied by a reaction rate constant as high as 0.38 min⁻^1^. By integrating experimental observations with finite element simulations, the contribution of the porous structure to the enhanced piezocatalytic activity was elucidated, offering new insights for the future development and application of piezocatalytic materials.

**Graphical abstract:**

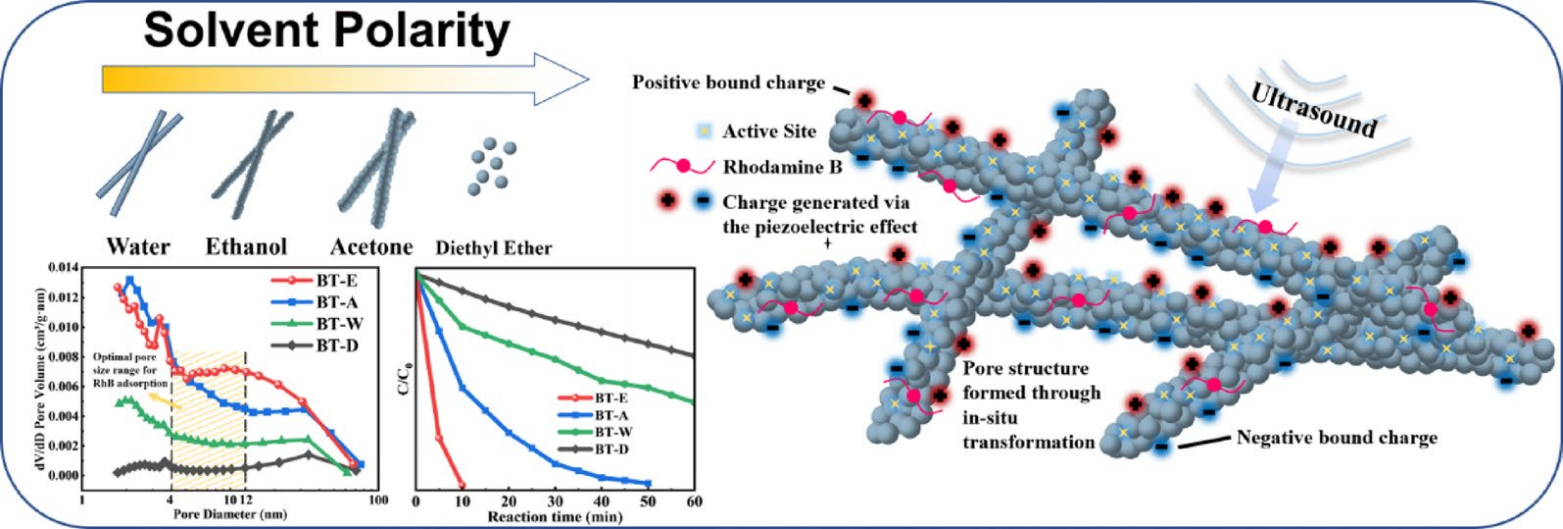

## Introduction

Amid growing global environmental concerns, the implementation of efficient environmental remediation strategies has become increasingly imperative. Taking dye wastewater treatment as an example, its high organic content and complex composition pose a substantial threat to the environment [1, 2]. Current wastewater treatment technologies primarily encompass physical, biological, and advanced oxidation processes (AOPs). Among these, physical methods merely transfer organic pollutants without fundamentally degrading them, usually resulting in secondary pollution [3]. Biological methods, in contrast, are constrained by microbial activity, limiting their practical applicability [4]. As a representative of AOPs, photocatalysis generates electrons and holes upon light irradiation, which interact with water to form highly oxidative reactive radicals, thereby effectively decomposing organic pollutants into harmless small molecules such as water and carbon dioxide. Although photocatalysis has been rapidly adopted due to its high efficiency, environmental friendliness, and operational safety, it still faces limitations, including the requirement for continuous light irradiation and low energy conversion efficiency [5].

Piezocatalysis, as an alternative AOP, utilizes bound charges generated on the surface of piezoelectric materials under mechanical stress to participate in redox reactions [6, 7]. Compared with photocatalysis, piezocatalysis offers the distinct advantage of harnessing localized mechanical energy—such as vibration, friction, or ultrasonic waves—to facilitate the chemical redox reactions. This reduces reliance on specific light sources, rendering it highly promising for environmental remediation. In piezocatalysis, the piezopotential generated on the catalyst surface plays a crucial role in determining catalytic activity. The piezopotential can be estimated by the equation [Eq. (1)]: where ${V}_{p}$ is the piezopotential, ${d}_{ij}$ is the piezoelectric charge coefficient,

1 $${V}_{p}=\frac{{d}_{ij}}{{\varepsilon }_{r}{\varepsilon }_{0}}\sigma w$$

${\varepsilon }_{r}$ is relative permittivity, ${\varepsilon }_{0}$ is permittivity of the free space, $\sigma$ is the applied stress and $w$ is the particle thickness. According to the [Eq. (1)], strategies to enhance the piezopotential include increasing the piezoelectric response of the material, reducing its dielectric constant, and amplifying mechanical strain. It is worth noting that although the equation indicates a positive correlation between particle size and piezopotential, the actual catalytic efficiency is also influenced by factors such as the effective exposure of the piezopotential and the specific surface area. An overly large particle size can adversely affect catalytic performance. Therefore, the design of piezocatalysts requires a comprehensive balance of these factors to optimize overall efficiency.

Barium titanate (BaTiO₃), as an environmentally friendly piezoelectric material, exhibits high polarizations, thereby improving charge separation efficiency and have been studied for piezocatalysis [8]. To improve its piezocatalytic performance, the efforts have been primarily focused on morphology engineering, ion doping, and surface modification. It has been reported that, compared to nanoparticles, BaTiO₃ nanowires exhibit a higher piezopotential along the polar axis [9]. Catalytic activity has also been significantly enhanced by adopting a strategy of Li/La ion doping into BaTiO₃ nanosheets, which additionally led to selective catalytic enhancement for specific pollutants [10]. Furthermore, the piezocatalytic performance can be improved by functionalizing the surface of BaTiO₃ nanoparticles with carboxyl groups [11].

It is important to note that the pore structure of catalysts plays a crucial role in catalytic processes by regulating specific surface area, mass transfer rate, selectivity, and stability, thereby enhancing catalytic efficiency. Nevertheless, in the field of piezocatalysis, research on pore structure modulation remains limited. It has been demonstrated that introducing 30% porosity into BaTiO_3_ ceramics improved the piezocatalytic degradation efficiency of methylene blue by 1.6 times, confirming that pore structure engineering can significantly enhance piezocatalytic performance [12]. Additionally, it has been found that the particle size of piezocatalysts significantly influences their catalytic performance; as particle size decreases, the number of exposed active sites increases, leading to enhanced adsorption capacity [13]. Therefore, nanoscale catalyst particles are highly desired in catalysis applications.

In the synthesis of nanocatalysts, wet-chemical methods [14] such as the sol–gel method [15], solvothermal method [16], co-precipitation method [17], and microemulsion method [18] are commonly employed to achieve smaller particle sizes. In these processes, factors such as solvent polarity, viscosity, and solubility influence the solubility of precursors, crystal growth rate, and interfacial energy, thereby regulating the morphology, pore structure, crystallinity, and surface functional groups of the material. Therefore, the appropriate selection of solvents can simultaneously optimize multiple properties of the catalyst, ultimately enhancing its catalytic performance.

In this study, BaTiO_3_ nanowires were selected as the research subject, and the solvothermal method was utilized to synthesize the nanocatalysts. By altering the reaction solvent, the pore structure of BaTiO_3_ nanowires was modulated, and their piezocatalytic activity was evaluated through the degradation of RhB solution. The effects of porous structures formed under different growth conditions on the crystal structure, piezoelectric response, and bulk conductivity were investigated, providing an innovative approach for the optimization of piezocatalysis.

## Experimental section

### Catalyst preparation

BaTiO_3_ nanowires were synthesized via a two-step solvothermal method. Initially, 0.4 mol/L tetrabutyl titanate was dissolved in 30 mL ethanol, ultrasonicated for 10 min, and stirred thoroughly to ensure uniformity, denoted as Solution A. Separately, a 10 mol/L NaOH solution was prepared in 30 mL and designated as Solution B. Under continuous stirring, Solution A was slowly added dropwise to Solution B to prevent the hydrolysis of tetrabutyl titanate. The resulting solution was then transferred into a 100 mL sealed autoclave and maintained at 180 °C for 12 h under continuous stirring. After the reaction, the flocculent precipitate was collected, washed thoroughly, and dried to obtain Na_2_Ti_3_O_7_ (NTO) nanowires. The synthesized NTO nanowires were then mixed with 0.1 mol/L Ba(OH)_2_·8H_2_O in four solvents—water, ethanol, acetone and diethyl ether—under vigorous stirring to ensure uniform dispersion. The second solvothermal reaction was carried out in a 100 mL sealed autoclave under continuous stirring. The precipitate obtained after the reaction was washed and dried to obtain the final product. The samples synthesized in water, ethanol, acetone, and diethyl ether are denoted as BT-W, BT-E, BT-A, and BT-D, respectively.

### Structural and morphological characterization

The phase composition of the catalysts was analyzed using X-ray diffraction (XRD, X’Pert PRO). The microstructure of the catalyst surface was observed with a field-emission scanning electron microscope (SEM, Zeiss Ultra Plus). High-resolution transmission electron microscopy (HRTEM), selected-area electron diffraction (SAED), and elemental distribution analyses were conducted using a field-emission transmission electron microscope (TEM, JEM-F200). Molecular structures were studied using a laser confocal Raman spectrometer (Raman, LabRAM Odyssey). The Brunauer–Emmett–Teller (BET) method was employed for specific surface area and pore structure analysis using a fully automatic surface area and porosity analyzer (ASAP 2460). The piezoelectric response of the catalyst was characterized using a piezoresponse force microscope (PFM, Bruker Dimension ICON).

### Piezocatalytic activity evaluation

Rhodamine B (RhB) was selected as the target pollutant to evaluate the piezocatalytic efficiency. In a typical test, 50 mg of catalyst powder was dispersed in 50 mL of RhB solution (5 mg/L) in a brown bottle to prevent light interference during the catalytic process. The solution was stirred for 1 h to achieve adsorption–desorption equilibrium between the catalyst and the dye solution. The brown bottle was then placed in a 100 W ultrasonic cleaner. At fixed intervals, 3 mL of the solution was extracted from the bottle, centrifuged, and the supernatant was analyzed using a UV–vis spectrophotometer (Lambda 750 S) to determine the UV absorption spectrum and evaluate the degradation efficiency.

The reusability of the catalyst was also assessed. After the reaction, the remaining dye solution was centrifuged, and the catalyst powder was thoroughly washed and dried. The recovered catalyst was subjected to repeated piezocatalytic activity tests following the same procedure as described above.

## Results and discussions

### Structure and morphology

**Fig. 1:**
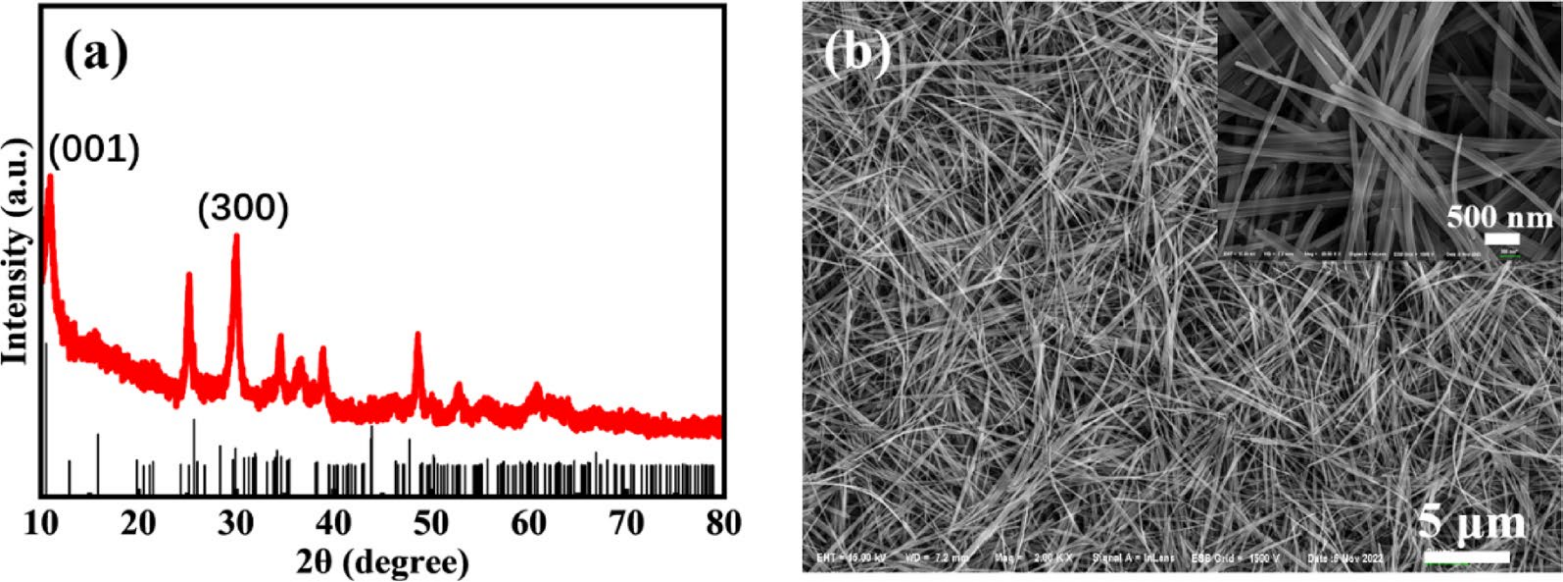
**a** XRD pattern and **b** SEM image of Na_2_Ti_3_O_7_ nanowires

The precursor was successfully synthesized by the first-step hydrothermal method, and its XRD pattern is shown in Fig. 1a. The strongest diffraction peaks at 2θ = 10.5° and 29.9° correspond to the (001) and (300) crystal planes of NTO (ICSD #01–070-9440), confirming the synthesis of NTO. The SEM image of the precursor (as shown in Fig. 1b) confirms the successful synthesis of NTO nanowires with a slender, uniform morphology and good dispersion. The average length of the NTO nanowires is approximately 13 μm, and the average diameter is about 71 nm, with the thinnest sections measuring as small as 32 nm (as shown in the inset of Fig. 1b).

**Fig. 2:**
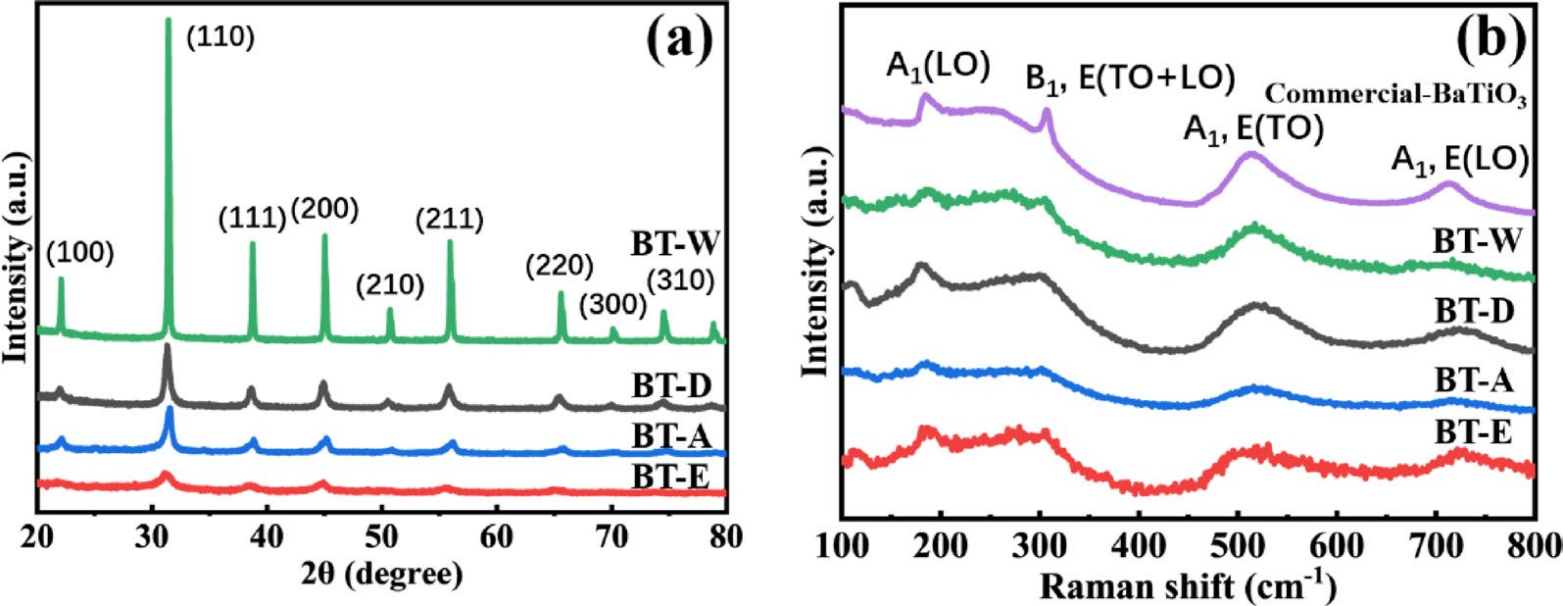
**a** XRD patterns and **b** Raman spectroscopy comparison of BaTiO_3_ nanomaterials prepared in four solvents

Figure 2a shows the XRD diffraction patterns of the precursor NTO nanowires after conversion to BaTiO₃ in the respective four solvents. The results confirm the successful synthesis of BaTiO₃ in all four solvents. Among them, BT-W exhibits the sharpest XRD peaks, indicating the highest crystallinity. In contrast, BT-E shows the lowest peak intensity and a certain degree of peak broadening. According to the relationship described by the Scherrer Equation [Eq. (2)]:

2 $$L=\frac{K\lambda }{\beta \mathit{cos}\theta }$$

where L is the crystallite size, β is the full width at half maximum (FWHM) of the peak, λ is the X-ray wavelength, and K is a shape factor, a smaller crystallite size reduces the number of planes contributing to diffraction, leading to broader and weaker peaks. The calculated crystallite sizes for BT-W, BT-E, BT-A, and BT-D are 58 nm, 5 nm, 18 nm, and 26 nm, respectively, which are generally follow the trend observed in the intensity of the diffraction peaks. Additionally, Raman spectroscopy revealed that BaTiO₃ synthesized in all four solvents exhibited characteristic tetragonal phase Raman peaks at 185, 306, 512, and 718 cm⁻^1^, as shown in Fig. 2b. Despite the nanoscale BaTiO₃ samples exhibiting pseudo-cubic characteristics in the XRD patterns, this apparent discrepancy arises from the fact that the long-range tetragonal distortion cannot be maintained at the nanoscale, and XRD only detects the average crystal structure. In contrast, Raman spectroscopy reflects lattice vibrations, and local Ti displacement still exists [19, 20]. XRD reveals the long-range order of the crystal, but due to the size confinement effect, when the crystallite size is reduced to the nanoscale, peak broadening causes the (002) and (200) reflections to overlap, making them difficult to resolve. Consequently, the characteristic diffraction peak near 45° appears as a single unsplit peak in the XRD patterns shown in Fig. 2a. To further investigate, the XRD patterns of BaTiO₃ synthesized in four different solvents were refined, and the results are shown in Table 1. R_wp_ refers to the weighted profile R-factor, which measures the deviation between the experimental diffraction pattern and the refined fitting curve. It is generally accepted that an R_wp_ value below 10% indicates a good fit. The calculated c/a values indicate that all samples exhibit a certain degree of tetragonality, with the BT-E sample showing the highest value of 1.005, confirming the coexistence of tetragonal and cubic phases across the synthesized nanomaterials [21, 22].

**Table 1:** Refinement results of XRD patterns for BaTiO₃ synthesized in four different solvents

Samples	a	b	c	c/a	R_wp_ (%)
BT-W	4.0099	4.0099	4.0195	1.002	7.51
BT-E	4.0415	4.0415	4.0632	1.005	7.23
BT-A	4.0054	4.0054	4.0192	1.003	9.31
BT-D	4.0406	4.0406	4.0448	1.001	7.92

**Fig. 3:**
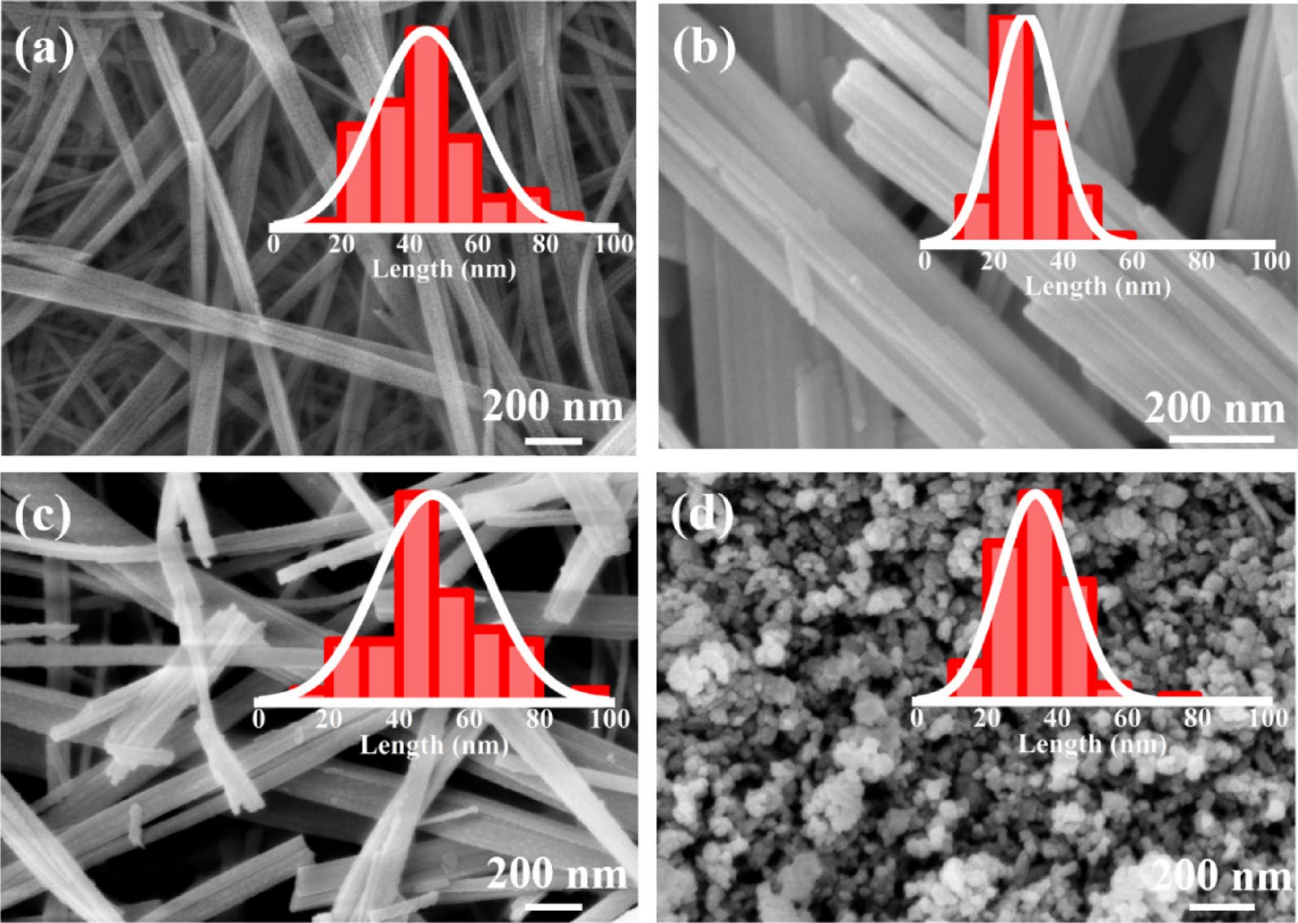
SEM comparison of **a** BT-W, **b** BT-E, **c** BE-A and **d** BT-D

The SEM images of BaTiO_3_ synthesized in four different solvents are shown in Fig. 3a–d. Uniform BaTiO_3_ nanowires were successfully obtained in water, ethanol and acetone solvents for BT-W, BT-E, BT-A samples, respectively. In contrast, the BT-D sample synthesized in diethyl ether exhibited a nanoparticle morphology rather than a nanowire structure. The average diameters of the nanowires and particle size are provided inside the figures.

This difference can be attributed to the weak polarity of diethyl ether, which is less effective than polar solvents like water in facilitating ion migration and diffusion [23, 24]. This reduction in reaction kinetics hinders the maintenance of the titanium-oxygen framework, leading to multidimensional crystal growth and the formation of isotropic nuclei. The formation of one-dimensional (1D) structures like nanowires typically requires lower surface energy to prevent crystal fracture or agglomeration. However, the low polarity and surface tension of diethyl ether are not conducive to the stability of 1D nanowires. During crystal growth, higher surface energy promotes fragmentation or contraction into particulate morphologies, which explains the formation of BaTiO₃ nanoparticles when diethyl ether is used as the solvent. This behavior reflects a distinct growth mechanism compared to the nanowire structures obtained in other solvents.

**Fig. 4:**
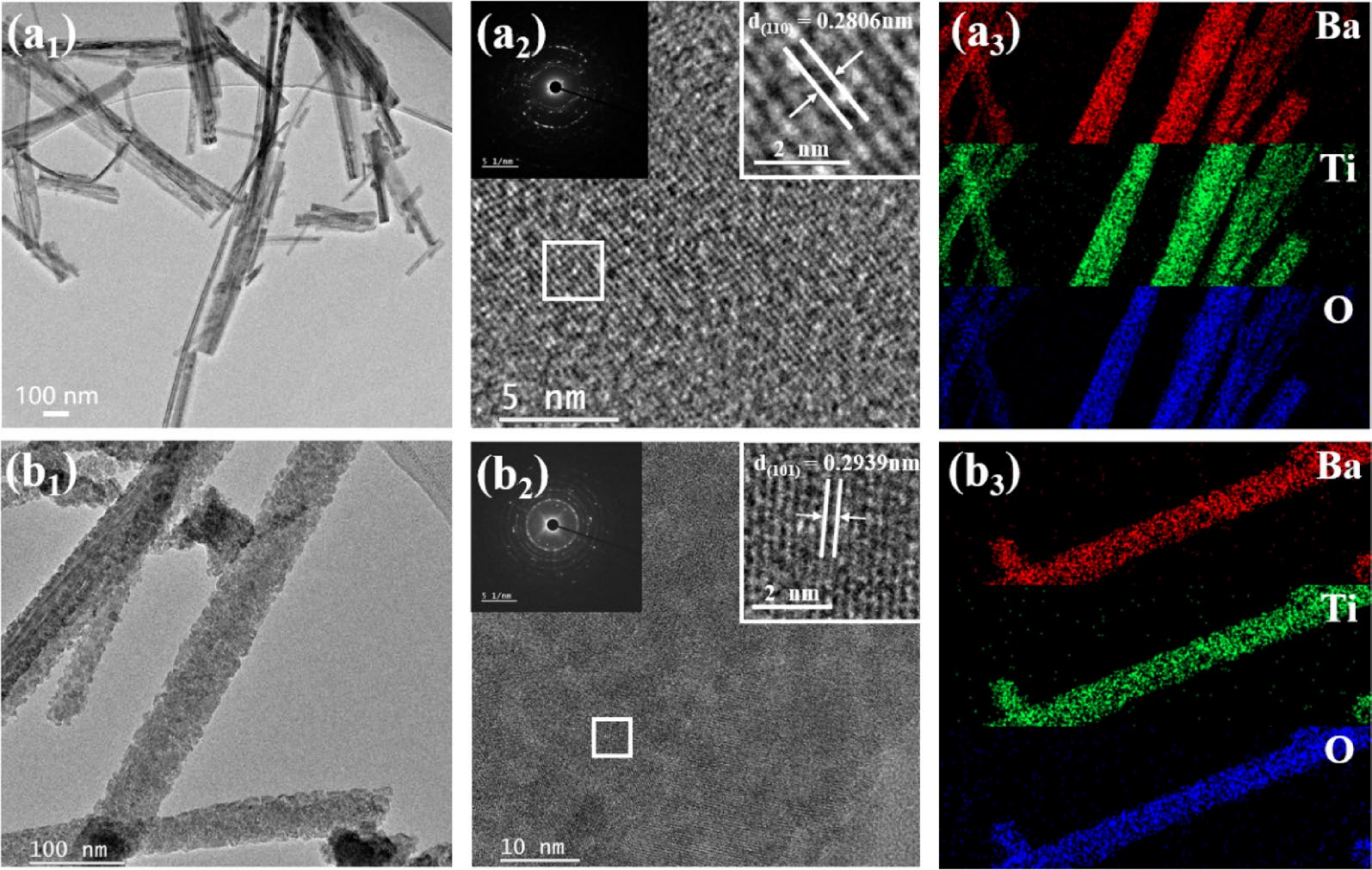
**a** and **b** show the TEM images of BT-W and BT-E, respectively. **a**_**1**_ and **b**_**1**_ are the corresponding HRTEM images; **a**_**2**_ and **b**_**2**_ show the lattice fringes of the two samples, while the insets present the SAED diffraction patterns and magnified views of the lattice fringes. **a**_**3**_ and **b**_**3**_ are the EDS spectra of BT-W and BT-E, respectively

As shown in Fig. 4a_1_–a_2_ and b_1_–b_2_, the HRTEM images of the BT-W and BT-E samples are presented. Unlike the BT-W nanowires shown in Fig. 4a_1_, which exhibit a smooth and dense morphology, Fig. 4b_1_ reveals that the BT-E nanowires are composed of loosely packed fine nanoparticles, forming a porous structure. This morphology supports the previously discussed crystallite size calculation for the BT-E nanowires. Although the average diameter measured via SEM is about 29 nm, the nanowires are actually composed of smaller nanoparticles at the microscopic level, which explains the smaller crystallite size derived from the XRD results.

Higher magnification TEM images in Fig. 4a_2_ and b_2_ reveal that BT-W displays well-defined lattice fringes, indicating good crystallinity, whereas BT-E exhibits a mixed microstructure of nanocrystalline and amorphous regions. This observation is consistent with the XRD results in Fig. 1, further supported by the SAED pattern (the upper left insets of Fig. 4b_2_), which shows slightly diffuse diffraction rings, confirming the coexistence of nanocrystalline and amorphous phases [25]. The upper right insets of Fig. 4a_2_ and b_2_ show the magnified lattice fringes of BT-W and BT-E, respectively. The measured lattice spacings are 0.2806 nm and 0.2939 nm, corresponding to the (110) and (101) crystal planes of BaTiO₃. Finally, Fig. 4a_3_ and b_3_ show the EDS elemental mapping of Ba, Ti, and O in BT-W and BT-E. The uniform distribution of these elements along the nanowires confirms the successful synthesis of stoichiometric BaTiO₃ in both samples.

### Underlying mechanism for BaTiO_3_ nanowires growth

When water is used as the solvent, previous studies have confirmed that the crystal growth follows a "dissolution–recrystallization" mechanism [26, 27]. The precursor dissolves in the alkaline environment, releasing soluble titanate ions that react with Ba^2^⁺ to form BaTiO_3_. The reaction can be represented as follows [Eq. (3)]:

3 $$ {\text{Na}}_{{2}} {\text{Ti}}_{{3}} {\text{O}}_{{7}} + {\text{3Ba}}^{{{2} + }} + {\text{6OH}}^{ - } \to {\text{3BaTiO}}_{{3}} + {\text{2NaOH}} + {\text{2H}}_{{2}} {\text{O}} $$

Under this mechanism, the diffusion rate of soluble reactants is high, and the nucleation rate is comparable to the growth rate, resulting in a fully developed growth process and larger crystallite sizes. This leads to a denser product with higher crystallinity, and the diameter of the BaTiO₃ nanowires becomes larger than that of the original NTO nanowires [28].

**Fig. 5:**
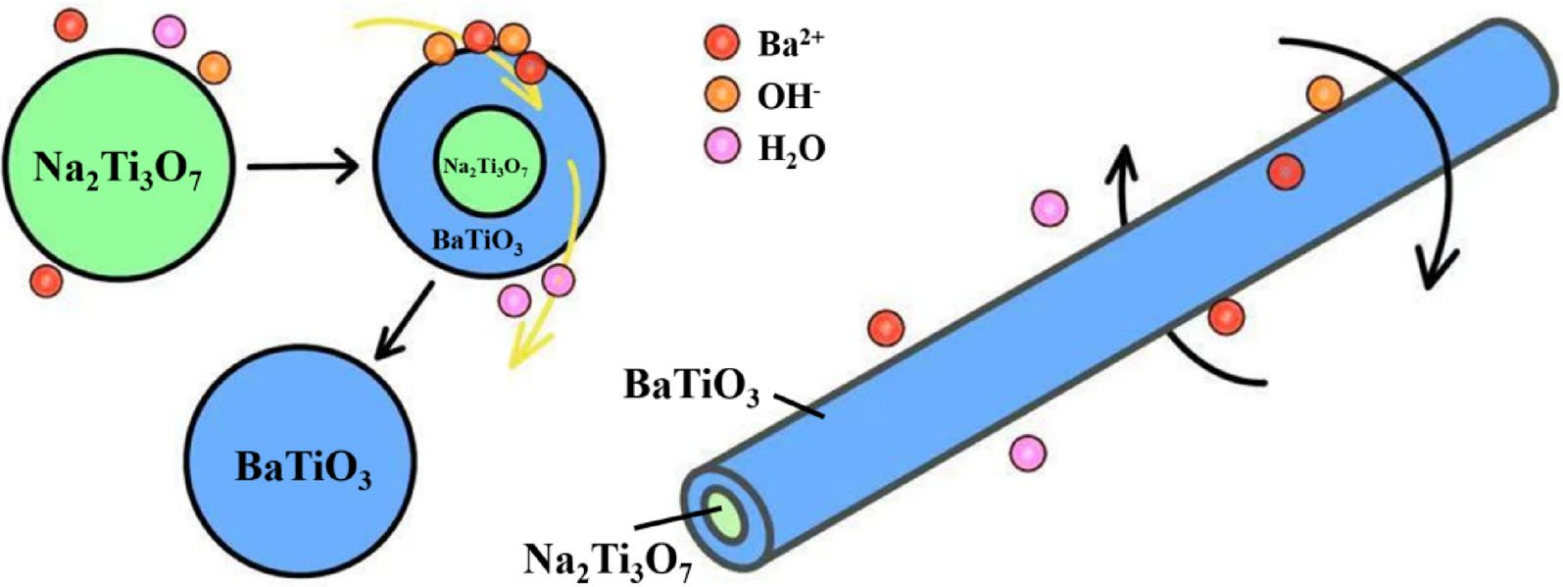
The schematic sketch of the in situ transformation mechanism

In contrast, when ethanol is used as the solvent, due to the extremely low solubility of the reactants, the "dissolution–recrystallization" mechanism is no longer applicable. Instead, Ba^2^⁺ ions, dissolved from the crystallization water of Ba(OH)₂·8H₂O, attach to the surface of the NTO nanowires. Owing to the relatively open structure of the TiO₆ octahedra, the material exhibits excellent ion-exchange properties. At this point, Ba^2^⁺ ions continuously penetrate from the surface into the NTO nanowires, reacting while preserving the original morphology of the nanowires and forming BaTiO₃ nanowires, as shown in Fig. 5. This process can be described as an in situ transformation mechanism [29].

Specifically, during the growth of BaTiO₃ crystals, due to the extremely low solubility of reactants in ethanol, the solution near the surface of NTO nanowires remains in a supersaturated state. Under supersaturation conditions, the relative consumption rate of supersaturated species for nucleation is much higher than that required for crystal growth [30]. After Ba^2^⁺ exchanges with Na⁺, BaTiO₃ crystals nucleate at a very high rate. However, as the reactants in the local microregion on the NTO surface become depleted and the concentration drops from supersaturation to saturation, the growth rate of BaTiO₃ crystals slows down. Under stirred hydrothermal reaction conditions, the reactants near the NTO nanowire surface are replenished in time, allowing Ba^2^⁺ to penetrate through micropores in the product layer into the interior under the combined effects of temperature and pressure, thus sustaining the reaction. This rapid nucleation process results in smaller BaTiO₃ crystal sizes, as the solution repeatedly cycles between supersaturated and saturated states in local regions, slowing down the growth rate and preventing a significant increase in the size of nucleated crystals over time, as confirmed by the XRD results in Fig. 1.

Furthermore, as shown in Fig. 4b_1_, BT-E exhibits a loose and porous structure, which can be well explained by considering the polarity and dielectric constant of the solvents. The polarity and dielectric constant of the solvent determine the dissociation degree of Ba(OH)_2_·8H₂O and the efficiency of ion transport, thereby influencing the reaction pathway from NTO nanowires to BaTiO_3_, including the nucleation and growth modes. In highly polar aqueous solutions (ε_r_ ≈ 80), Ba^2+^/OH^−^ are sufficiently supplied and diffuse uniformly into the nanowires, resulting in the formation of dense-surfaced and highly crystalline BaTiO_3_ nanowires through a dissolution–recrystallization mechanism. In contrast, in ethanol (ε_r_ ≈ 25) and acetone (ε_r_ ≈ 21), limited solubility and ion transport restrict the reaction, leading to preferential nucleation on the outer surface and an imbalance in inward and outward diffusion rates (with Na^+^ diffusing outward faster than Ba^2+^ diffuses inward). This induces the formation of a rough and porous structure via the Kirkendall effect, accompanied by reduced crystallinity. In low-polarity diethyl ether (ε_r_ ≈ 4), the precursor exhibits extremely low solubility, and BaTiO_3_ nuclei can only form in localized microenvironments under high-temperature and high-pressure conditions. Due to the high surface energy of crystals in such low-polarity solvents, the system tends to minimize its total energy by favoring isotropic growth, ultimately resulting in the formation of nanoparticles.

### Pore structure of BaTiO_3_ nanowires

As shown in Table 2, the specific surface area and porosity of BaTiO₃ prepared in four different solvents were measured, among which the biggest surface area and pore volume with the lowest pore diameter was obtained for BT-E sample. A larger specific surface area generally enhances catalytic activity and efficiency by providing more active sites. However, the actual catalytic performance also depends on the pore structure of the material.

**Table 2:** Specific surface area and porosity of BaTiO_3_ prepared in four solvents

Samples	BET surface area (m^2^/g)	Pore volume (cm^3^/g)	Pore diameter (nm)	
BT-E	115.44	0.44		11.83
BT-A	81.36	0.27		15.97
BT-W	43.49	0.18		18.23
BT-D	14.24	0.10		30.26

**Fig. 6:**
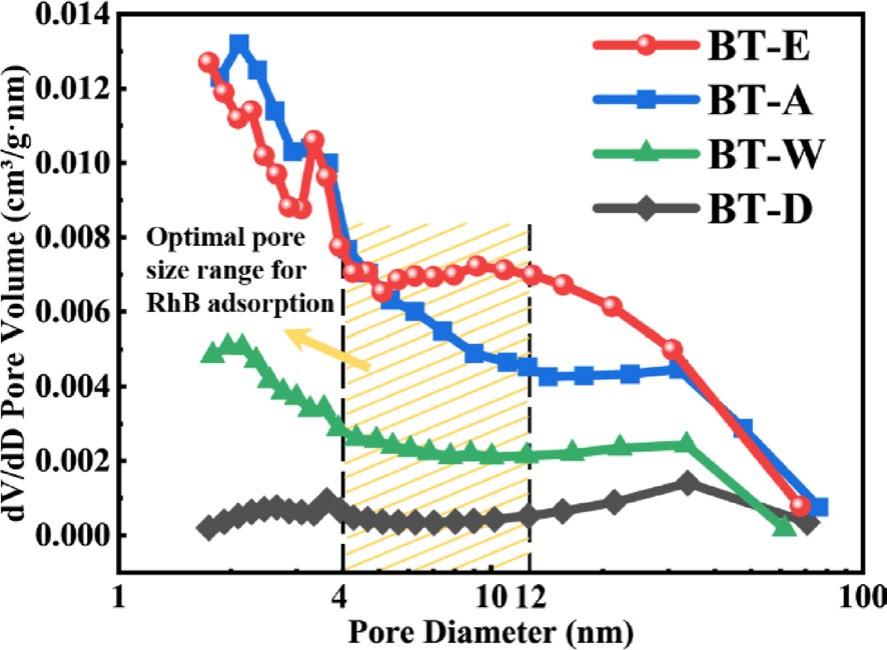
Pore size distribution of BaTiO_3_ prepared in four different solvents

As shown in Fig. 6, the pore size distributions of BaTiO₃ synthesized in four different solvents are presented. The y-axis represents the proportion of pore volume within different pore size ranges, reflecting the contribution of each pore size range to the total pore volume. Micropores (< 2 nm) contribute significantly to increasing the specific surface area, whereas mesopores (2–50 nm) represent an optimal balance between surface area and molecular diffusion/adsorption performance. The molecular size of Rhodamine B is approximately 1.8 nm [31], considering that its hydrated diameter is slightly larger than its molecular size, the nanopore size should exceed 2 nm to ensure proper adsorption and transport of dye molecules. It is studied that for porous catalysts, an optimal pore size-to-dye molecule size ratio of 2–6 is favorable for enhancing adsorption efficiency [32]. For the BT-E and BT-A samples, although the proportion of pore sizes in the 2–4 nm range is relatively similar, BT-E shows a significantly higher proportion of pores in the 5–12 nm range compared to BT-A, and therefore is expected to exhibit higher adsorption and transport efficiency for Rhodamine B molecules.

**Fig. 7:**
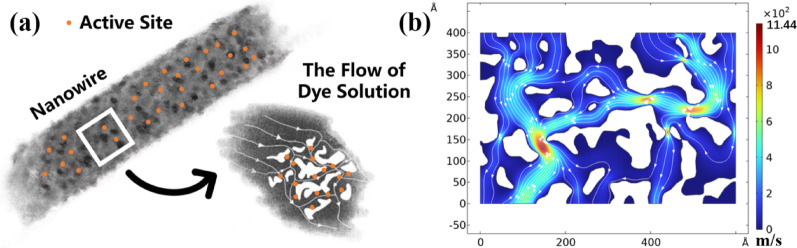
**a** The schematic diagram of dye solution traversing nanowire pores. **b** The simulation of fluid flow through the pores of BaTiO₃ nanowires

A combination of a high specific surface area and well-designed pore structure reduces diffusion limitations, allowing reactant and product molecules to flow smoothly within the catalyst. As indicated in Table 2, BT-E exhibits the highest specific surface area, the largest pore volume, and the smallest average pore size compared to the other samples. Pore volume refers to the total volume of pores within the material and reflects the amount of usable pore space per unit mass or volume. A larger pore volume allows for the adsorption and accommodation of more reactants during the catalytic process. Meanwhile, as shown in Fig. 7a, the small pore size on the nanowire surface enables the dye solution to flow through the nanowire pores, interacting with more active sites rather than merely making simple contact with the nanowire surface.

To investigate the relationship between pore structure and solution transport efficiency, the COMSOL Multiphysics software package was used to simulate the interaction between solution transport and pore structure based on the finite element method (FEM). Studies have shown that ultrasonic cavitation can generate microjets in liquids with velocities reaching up to 110 m/s, typically with diameters exceeding 1 μm and lengths of several micrometers [33]. A model with dimensions of 600 Å × 400 Å was extracted from the nanowire, with the model width precisely matching the nanowire diameter. It was assumed that the cavitation bubble collapses above the model, generating a microjet that travels downward through the structure, while fluid flow within the pores does not penetrate the solid material. Given the extremely short duration of the cavitation microjet impact (~ 10⁻⁹ s), the external conditions can be considered unchanged during the impact process. Considering the nanoscale size of the pores, slip boundary conditions were applied to solve the Navier–Stokes equation.

The simulation results, shown in Fig. 7b illustrate the velocity field with streamlines. It can be observed that after the microjet generated by ultrasonic cavitation enters the pores on the BaTiO_3_ nanowires, the flow velocity becomes extremely high in the narrower channels, with the instantaneous speed reaching up to 1144 m/s, which greatly accelerates the mass transfer rate of dye molecules. The increased flow velocity enables dye molecules to reach the BaTiO_3_ surface more rapidly, effectively thinning the diffusion boundary layer and increasing the concentration of reactants at the surface. This not only ensures the full utilization of active sites but also reduces the recombination probability of screening charges, thereby significantly enhancing the piezocatalytic efficiency.

### Piezoelectric response

**Fig. 8:**
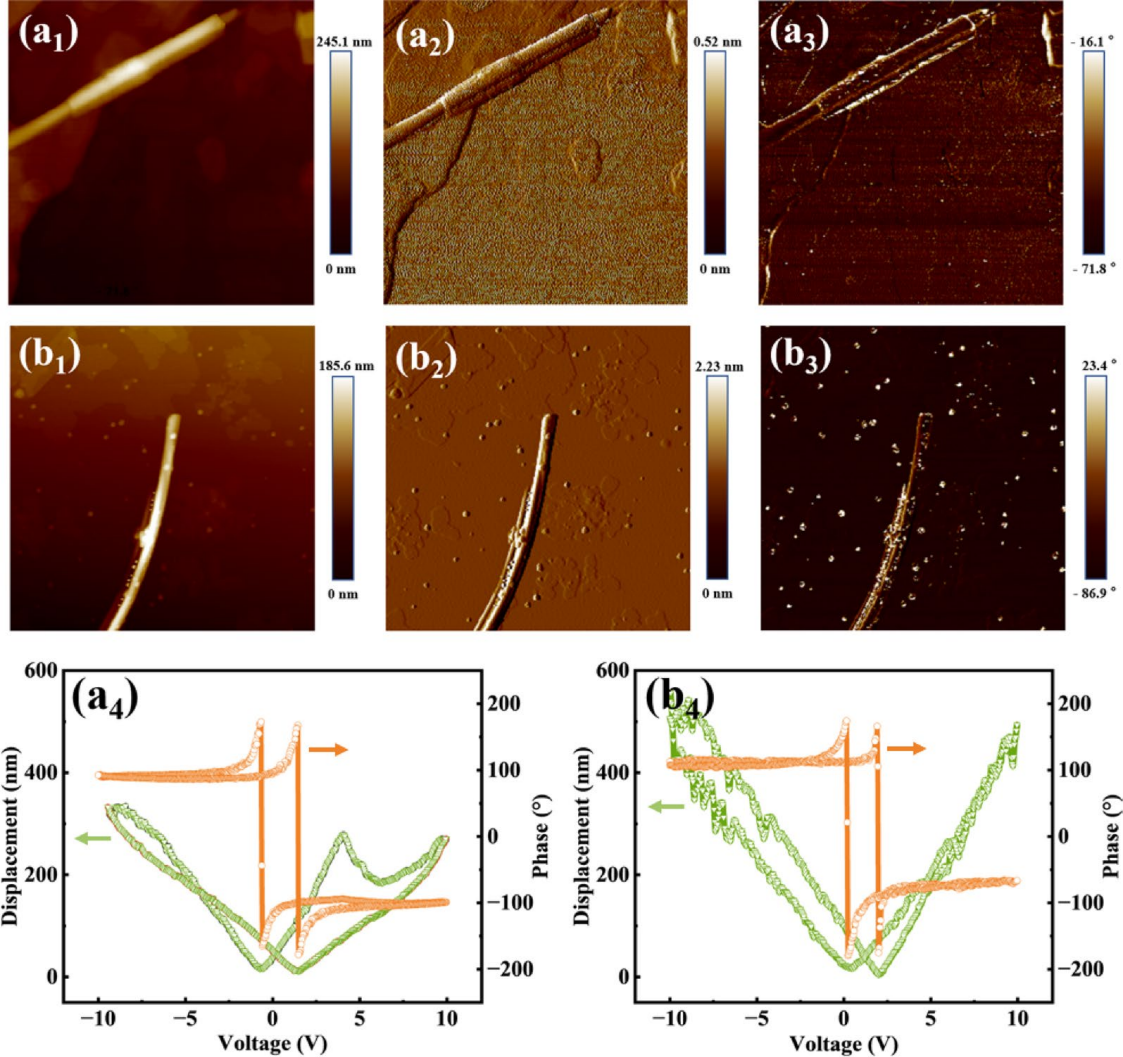
**a**_**1**_–**a**_**3**_ and **b**_**1**_–**b**_**3**_ are the morphology image, amplitude image, and phase image of BT-W and BT-E, respectively; **a**_**4**_ and **b**_**4**_ show the hysteresis loops of BT-W and BT-E

To investigate the effect of solvent on the piezoelectric performance of BaTiO₃, BT-W and BT-E were characterized by PFM measurements, as shown in Fig. 8. Figure 8a_1_–a_3_ and b_1_–b_3_ display the morphology, amplitude, and phase images of BT-W and BT-E, respectively. As shown in Fig. 8a_4_ and b_4_, under the same applied electric field, the displacement variation of BT-E is approximately twice that of BT-W, indicating a stronger piezoelectric response, which can be attributed to the higher structural flexibility of smaller grains [34]. With decreasing particle size, nanoparticles are able to withstand and recover from much larger strains. Moreover, the Young’s modulus decreases significantly with size reduction, indicating that the material deforms more easily under external force, thereby amplifying the piezoelectric response. According to the [Eq. (1)], BT-E is therefore expected to exhibit enhanced piezocatalytic performance. In addition, the inhomogeneity during the growth process in ethanol leads to a rough and porous structure on both the surface and interior of the nanowires, which further contributes to an increase of the piezopotential. Specifically, the piezopotential originates from lattice strain under external mechanical force, and the presence of pores causes local strain concentration or gradient variation, resulting in a non-uniform stress field. This stress field amplifies local lattice deformation and enhances polarization, thereby increasing the piezopotential. In addition, the porous structure introduces regions of low dielectric constant (as pores can be approximated as air), which reduces the overall relative permittivity of the material and further boosts the piezopotential [35].

Additionally, in Fig. 8b_4_, the butterfly curve exhibits an offset from the zero-point position, suggesting the presence of an internal electric field within the BT-E [36]. It was concluded that the formation of pores modifies the microstructure in a way that significantly increases the piezopotential, thus reinforcing the internal electric field [37]. The existence of this internal electric field effectively stabilizes the polarized charges and enhances the screening charge effect, thereby improving the catalytic efficiency [38]. Furthermore, the internal electric field can also enhance the surface adsorption capacity for specific reactants, thereby lowering the reaction activation energy and further improving catalytic performance.

### Piezocatalytic performance

**Fig. 9:**
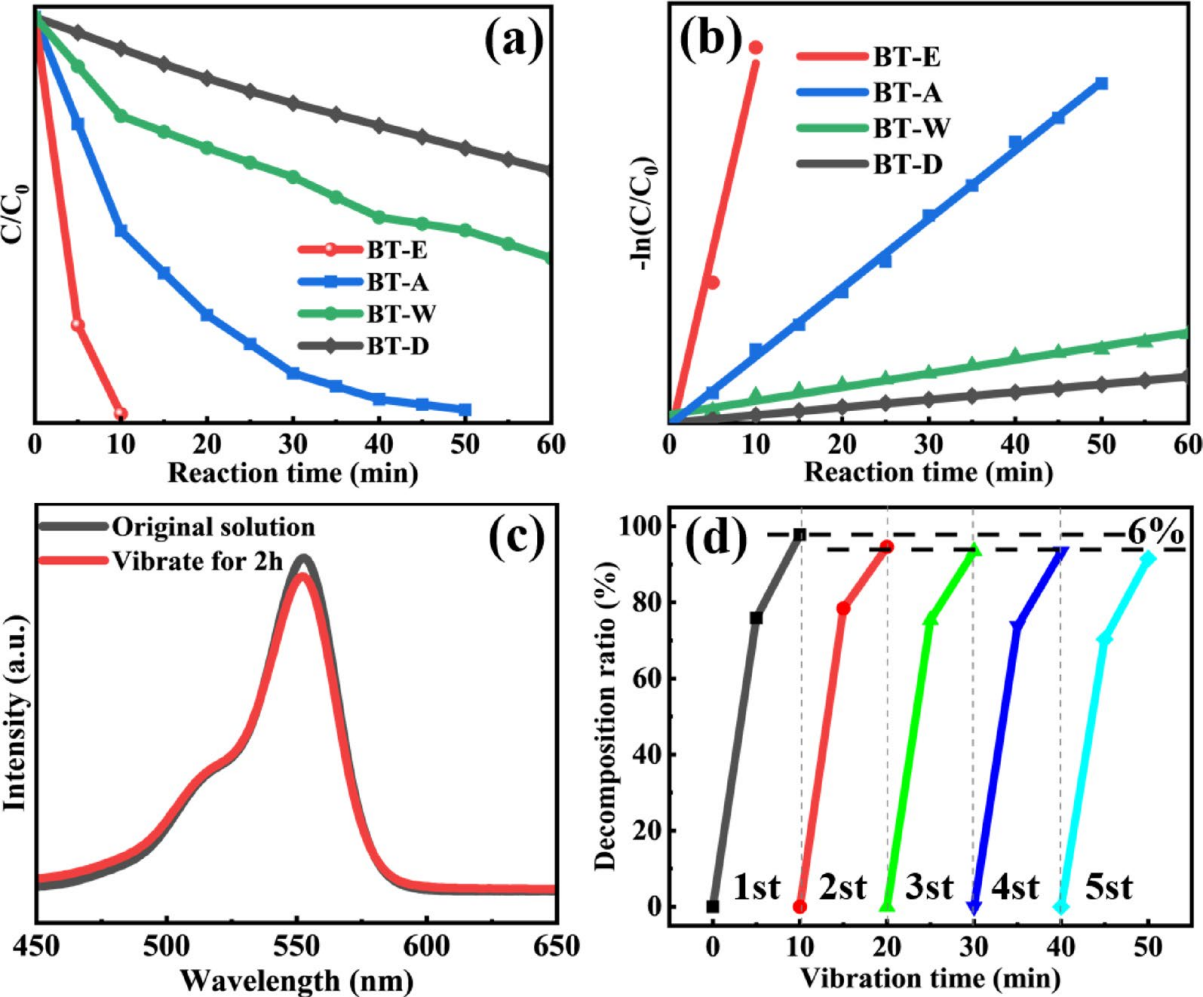
**a** Degradation rate and **b** Catalytic reaction rate constant of catalysts synthesized in four solvents; **c** Comparison of solution absorbance before and after ultrasonic treatment; **d** Degradation rate variation over five catalytic cycles

As shown in Fig. 9a, the piezocatalytic performance of BaTiO₃ synthesized in four different solvents is presented, where C represents the dye solution concentration at reaction time t (min), and C₀ represents the initial concentration of the dye solution. It can be observed that BT-E exhibited exceptional piezocatalytic performance, achieving a RhB dye degradation efficiency of up to 98% within 10 min under ultrasonic vibration. In contrast, the catalytic performance of BT-A, BT-W and BT-D gradually decreased. The reaction rate constant k for the piezocatalytic process was calculated using the following equation [Eq. (4)] [39]:

4 $$\text{In }\frac{\text{C}}{{C}_{0}} = -\text{kt}$$

The calculated piezocatalytic reaction rate constant for BT-E is approximately 0.38 min⁻^1^, while the rate constants for BT-A, BT-W and BT-D are 0.069 min⁻^1^, 0.014 min⁻^1^, and 0.0078 min⁻^1^, respectively. The corresponding graphical representation is shown in Fig. 9b.

To eliminate potential interference from ultrasonic vibration alone, control experiments were conducted without the addition of a catalyst. As shown in Fig. 9c, the dye concentration showed only a slight decrease after 2 h, confirming that the catalytic effect was not due to ultrasound. The reusability of the catalyst was also assessed using BT-E. As shown in Fig. 9d, the degradation efficiency decreased by only 6% after five cycles of piezocatalytic tests, which can be attributed to unavoidable minor losses during catalyst recovery. This result confirms the sustainability and stability of the catalyst for repeated use in piezocatalytic applications.

### Mechanism analysis of enhanced piezocatalytic performance

**Fig. 10:**
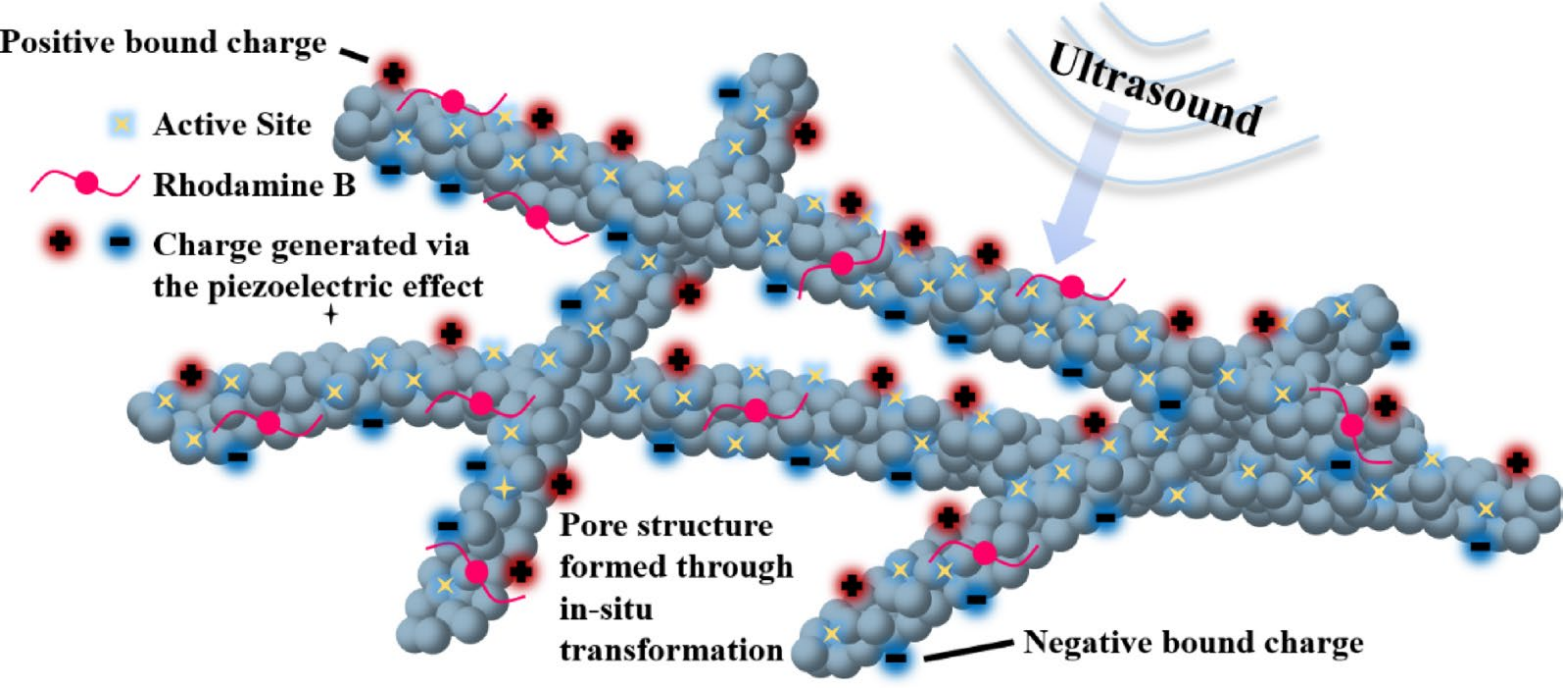
Schematic diagram of Enhanced Piezocatalytic Performance

It is observed that BT-E exhibits significantly superior piezocatalytic performance, as illustrated in Fig. 10. Based on existing experimental data, this enhancement is closely related to the transformation mechanism of NTO nanowires into BaTiO₃ in different solvents. When ethanol is used as the solvent, the solubility of the reactants is extremely low, rendering the "dissolution–recrystallization" mechanism inapplicable. Instead, the transformation follows an in situ conversion mechanism. In this process, Ba^2^⁺ ions attach to the surface of NTO nanowires and, leveraging the excellent ion-exchange properties of the TiO₆ octahedra, continuously penetrate from the outer surface into the interior of the nanowires, reacting and forming BaTiO₃ nuclei while maintaining the original nanowire morphology. This outward-to-inward reaction leads to the formation of a loose and porous structure on the nanowire surface, consistent with the porosity measurements in Table 2. Furthermore, the pore structure formed under such non-uniform growth conditions induces local strain and introduces low-dielectric-constant pores. The synergistic effect of these two factors enhances the piezopotential of the catalyst.

Due to this unique growth and crystallization mechanism, BT-E exhibits a superior pore structure, characterized by a larger pore volume, smaller pore size, and a more favorable pore size distribution for the adsorption of specific reactants (RhB). A larger pore volume facilitates the adsorption and accommodation of reactants, while smaller pore sizes ensure that the dye solution interacts with the nanowires beyond simple surface contact. Under ultrasonic cavitation, the microjets passing through the pores of the nanowires achieve higher flow velocities. This not only ensures the full utilization of active sites but also increases the surface reactant concentration, thereby reducing the recombination probability of screening charges and significantly enhancing the piezocatalytic efficiency.

Moreover, a larger pore volume reduces conductive pathways within the catalyst, which macroscopically manifests as an increase in resistivity. Electrical resistivity measurements indicate that BT-W and BT-E exhibited resistivities of approximately 4.3 × 10^8^ Ω·cm and 1.9 × 10^9^ Ω·cm, respectively, with the latter being three times higher. According to the "screening charge effect" mechanism [40], higher bulk resistivity minimizes the migration and dissipation of surface screening charges into the material bulk. This promotes the retention of screening charges on the catalyst surface, ultimately improving catalytic efficiency.

## Conclusion

BaTiO₃ nanowires were synthesized via a solvothermal method, with the catalyst's pore structure regulated by varying the solvent. The optimized sample exhibited high piezocatalytic activity, achieving a 98% degradation rate of RhB solution within 10 min and a reaction rate constant of 0.38 min⁻^1^. Through experimental results and finite element simulations, the contribution of porous structures to the enhanced performance of piezocatalysts was elucidated. The porous structure not only improved the transport rate of reactants but also enhanced the internal electric field and increased the bulk resistivity of the material. These factors effectively facilitated the participation of screening charges on the catalyst surface in redox reactions, significantly boosting the piezocatalytic efficiency. This study provides valuable insights into the design and optimization of piezocatalysts for environmental remediation applications.

## Data Availability

The datasets used and/or analysed during the current study are available from the corresponding author on reasonable request.
